# Computational Studies on Microreactors for the Decomposition of Formic Acid for Hydrogen Production Using Heterogeneous Catalysts

**DOI:** 10.3390/molecules28145399

**Published:** 2023-07-14

**Authors:** Eleana Harkou, Panayiota Adamou, Kyproula Georgiou, Sanaa Hafeez, Sultan M. Al-Salem, Alberto Villa, George Manos, Nikolaos Dimitratos, Achilleas Constantinou

**Affiliations:** 1 Department of Chemical Engineering, Cyprus University of Technology, 57 Corner of Athinon and Anexartisias, Limassol 3036, Cyprus; ea.harkou@edu.cut.ac.cy (E.H.); paa.adamou@edu.cut.ac.cy (P.A.); ks.georgiou@edu.cut.ac.cy (K.G.); 2 School of Engineering and Materials Science, Queen Mary University of London, London E14NS, UK; sanaa.hafeez@qmul.ac.uk; 3 Environmental & Life Sciences Research Centre, Kuwait Institute for Scientific Research, P.O. Box 24885, Safat 13109, Kuwait; ssalem@kisr.edu.kw; 4 Dipartimento di Chimica, Universitá degli Studi di Milano, via Golgi, 20133 Milan, Italy; alberto.villa@unimi.it; 5 Department of Chemical Engineering, University College London, London WCIE7JE, UK; g.manos@ucl.ac.uk; 6 Dipartimento di Chimica Industriale “Toso Montanari”, Alma Mater Studiorum Università di Bologna, Viale Risorgimento 4, 40136 Bologna, Italy; nikolaos.dimitratos@unibo.it; 7 Center for Chemical Catalysis-C3, Alma Mater Studiorum Università di Bologna, Viale Risorgimento 4, 40136 Bologna, Italy

**Keywords:** hydrogen, formic acid, dehydrogenation, microreactor, membrane, carbon monoxide

## Abstract

Sustainable alternatives to conventional fuels have emerged recently, focusing on a hydrogen-based economy. The idea of using hydrogen (H_2_) as an energy carrier is very promising due to its zero-emission properties. The present study investigates the formic acid (FA) decomposition for H_2_ generation using a commercial 5 wt.% Pd/C catalyst. Three different 2D microreactor configurations (packed bed, single membrane, and double membrane) were studied using computational fluid dynamics (CFD). Parameters such as temperature, porosity, concentration, and flow rate of reactant were investigated. The packed bed configuration resulted in high conversions, but due to catalyst poisoning by carbon monoxide (CO), the catalytic activity decreased with time. For the single and double membrane microreactors, the same trends were observed, but the double membrane microreactor showed superior performance compared with the other configurations. Conversions higher than 80% were achieved, and even though deactivation decreased the conversion after 1 h of reaction, the selective removal of CO from the system with the use of membranes lead to an increase in the conversion afterwards. These results prove that the incorporation of membranes in the system for the separation of CO is improving the efficiency of the microreactor.

## 1. Introduction

A major contributor to climate change is the combustion of large quantities of fossil fuels in the energy sector. Therefore, providing sustainable alternatives to conventional fuels is gaining attention in industrial circles and becoming mandatory in scientific fields of research. All of which are attempting to stabilize the harmful environmental effects of fossil fuels [1]. The key is to find a back-up source that is secure, renewable, and internationally available. Hydrogen (H_2_) is a naturally abundant, clean, and renewable energy source that can be exploited as an energy carrier and a potential transportation fuel. It is an emission-free energy carrier and can be produced through numerous paths such as thermochemical (TC) and biological processes, electrolysis, and photolysis [2]. The transition, from a fossil fuel-based economy to a hydrogen-market faces many technical and socioeconomic hurdles that hinder the implementation of H_2_ as a future energy source [3].

Instead of H_2_, liquid organic hydrogen carriers (LOHCs) can be utilized. Formic acid (FA/CH_2_O_2_) is a great example of a LOHC. It can be generated by the hydrogenation of carbon dioxide (CO_2_), and its decomposition takes place in mild conditions. It is a safe option to transport and store when compared with H_2_. FA can contain up to 4.4 wt.% of H_2_, which is double than the content of compressed H_2_ at a pressure of 350 bar at the same volume, and an energy density of 2.1 kWh/L [4]. FA can be decomposed via two pathways as depicted in Equations (1) and (2). The first, which is the more desirable reaction, includes the decarboxylation towards CO_2_ and H_2_. The second pathway, the undesirable route, is the decarbonylation towards carbon monoxide (CO) and water (H_2_O) and must be avoided due to the formation of CO, which poisons and deactivates the catalyst [5]. The co-product, CO_2_, of the decomposition of FA can be converted back into FA, either electrochemically or through catalytic hydrogenation, promoting a circular energy economy with net-zero CO_2_ emissions released into the atmosphere [6,7]. In addition, FA can be derived from plant biomass processing, promoting renewability of the H_2_ from this technology with significant developments in the recent years for the sustainable production of FA from lignocellulosic biomass [8].

(1) $$\mathrm{HCOOH}(l)\;\to {\mathrm{CO}}_{2}+H_{2}$$

(2) $$\mathrm{HCOOH}(l)\;\to \mathrm{CO}+H_{2}O$$

CO binds strongly with the metal center of catalysts causing a good fraction of it to deactivate. It also poisons Proton Exchange Membranes (PEM) of fuel cells [9,10]. The undesirable CO formation can be limited by the appropriate selection of suitable catalysts. Heterogeneous and homogenous catalysts can be used for the decomposition of FA, with the former being preferable. Homogeneous catalysts are more stable and are an option that is hard to separate from the mixture and reuse it. Conversely, heterogeneous catalysts offer various advantages including reusability, recyclability, and that they can be shaped into different geometries [11,12]. Among the heterogeneous catalysts investigated for the decomposition of FA, the Pd- and Au-based catalysts as well as their bimetallic materials are considered the most effective materials for this reaction [13,14], favoring the production of H_2_ against the formation of CO [15]. Choi et al. [16] developed palladium–silver (Pd.Ag) alloy cores with thin Pd shells as a catalyst for efficient H_2_ production from FA decomposition in an aqueous solution. The catalytic activity of PdAg@Pd ONCs toward FA decomposition was optimal, achieving at 50 °C a turnover frequency (TOF) of 21,500 h^−1^. Gas chromatography (GC) was used to detect CO contamination where the selective H_2_ generation was confirmed. Further studies on the catalyst disclosed that the atomic ratio of Pd/Ag and the atomic layer of Pd shells. The results clearly demonstrated that the approach of core–shell engineering is effective for the efficient development of catalysts to generate H_2_ from FA, with no contamination of CO. Zhang et al. [17] synthesized ruthenium (Ru) catalysts supported on two different porous organic polymers (POPs). Ru@POPs-TPP had a low activity and a significant amount of CO in the products (≈2.221%). The Ru@POPs-PPh3 catalyst exhibited an excellent activity with 7284 h^−1^ of TOF value. Even though the surface areas of the two catalysts were similar, their catalytic performance was significantly different. The Fourier transform infrared (FT-IR) spectra showed that the properties of ligands in the polymer framework may influence the overall performance of the catalysts.

Experimental data and density functional theory (DFT) analysis were combined by Barlocco et al. [18], in order to investigate the role of Au in enhancing the selectivity and activity of Pd catalysts for FA decomposition. Monometallic Pd and Au catalysts were also investigated and showed lower catalytic activities than Pd.Au alloy catalysts, confirming the superiority of a bimetallic catalytic system. The optimal performance was exhibited by the 1%PD_6_Au_4_@HHT catalyst (TOF 3539 h^−1^). Monometallic Pd catalyst was found to deactivate due to leaching and CO poisoning while 1%PD_6_Au_4_@HHT resulted in excellent stability after six continuous runs.

Over the years, different reactor types and set-ups were utilized for the catalytic decomposition of FA. The most common and studied systems are packed bed reactors, knowing that they are significant for heterogeneous catalysis [15,19,20]. Batch reactors were also evaluated for this reaction [21], but continuous stirred tank reactors were more preferable due to their ability to produce a stable and continuous stream of H_2_ for fuel cell implementations. Also, microchannel reactors have gained lot of attention due to their high efficiency and in situ H_2_ supply but still high-performance microreactors for FA decomposition are not developed for large-scale applications [22,23]. Membrane technology may also be used for the catalytic decomposition of FA. Membranes offer the ability to selectively separate different reaction components within the reactor. The introduction of a membrane into the continuous flow system can improve the performance of the reaction by removing the CO formed during the process. A membrane reactor can operate at the same time for reactions and separation of various components reducing significantly the cost of a process [24]. Recovery of products and purification of H_2_ can occur in a single unit providing the opportunity to improve efficiency and lower the cost. The most developed membranes for separating H_2_ are polymeric membranes due to their better permeability and mild operating conditions, and they have been already applied at a commercial scale. Another type of membranes are dense metal membranes, usually from palladium (Pd), nickel (Ni), platinum (Pt), and their alloys. Their structure allows the selective diffusion of H_2_ while rejecting other gases. Pd-based membranes are the most studied, but their high cost limits their application and practicality [25]. A tubular membrane reactor for the catalytic decomposition of ammonia (NH_3_) was employed by Cechetto et al. [26]. A Ru-based catalyst was used with double-skin Pd membranes. Results were compared with that of a packed bed reactor which indicated that the membrane enhances the overall performance of the reactor, achieving higher conversions. Above 425 °C, a conversion of 100% was observed with 86% H_2_ recovery of 99.998% purity.

Hafeez et al. [27] investigated a novel microreactor configurations for FA decomposition with the use of a 5 wt.% Pd/C catalyst. Computational methods were used to evaluate the experimental data. Results showed that the packed bed and coated wall microreactors achieved a comparable performance with each other, with the coated wall configuration being slightly superior. The membrane microreactor exhibited the best performance compared with other microreactors given the fact that the deactivation from CO formation was alleviated with the use of a membrane.

In previous works carried out by our group [20], considering the formic acid decomposition using the Pd/C catalyst, the investigation was firstly carried out in batch and continuous flow reactors where experimental and theoretical studies took place. The formation of CO and herein the deactivation of the catalyst due to poisoning were a challenge we tried to minimize to ppm level. Different reactor configurations considering continuous flow reactors were studied to eliminate the production of CO [27].

Main aim of the work is to investigate and study different parameters in order to enhance the conversion of formic acid and the removal of CO, which inhibits the reaction, by using membrane configurations and therefore to improve stability of the catalyst. In the present work, CFD analysis was conducted on a packed bed, single and double membrane microreactors for FA decomposition with the use of commercial 5 wt.% Pd/C catalyst offering for the first time a reasonable, effective solution on the efficient removal of CO. Process simulation modeling using computational fluid dynamics (CFD) is valuable for a reaction system as it gives significant information regarding various parameters on different reactor configurations that can be validated from experimental data [20,27,28,29,30,31,32,33].

## 2. Results and Discussion

### 2.1. Packed Bed Microreactor

**Figure 1:**
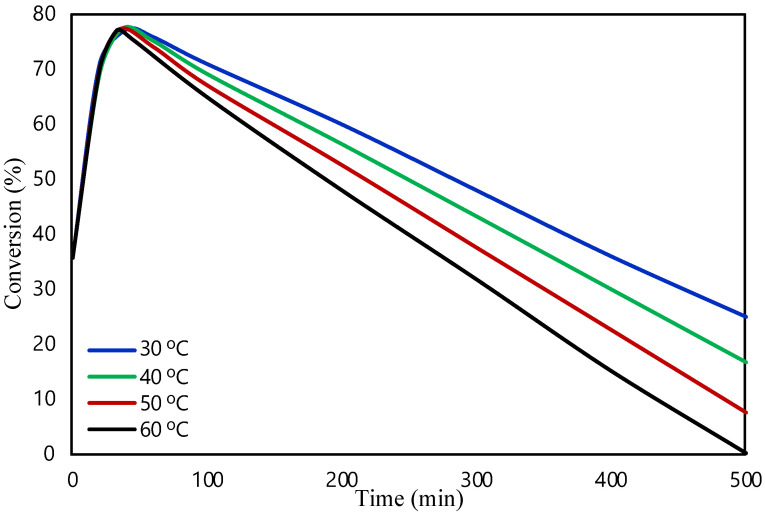
Temperature effect on FA conversion in packed bed microreactor.

In this section, the obtained results from the modelling of the packed bed are presented. The validity of the model was examined by our group in a previous study [20]. The FA reaction occurred at 1 bar of pressure and at an initial flow rate of 0.05 mL/min in the presence of Pd/C catalyst. The commercial Pd/C catalyst showed a 99.9% selectivity for H_2_, and the characterization and techniques used can be found in a previous work [34]. The small quantity of CO produced is significant to affect the stability of the catalyst, as it leads to the poisoning of the catalyst. Figure 1 shows the effect of temperature on the FA conversion. The conversion of FA is determined as the mass balance of FA in the reactor ((F_FA,in_ − F_FA,out_)/F_FA,in_). The FA reaction was investigated at 30, 40, 50, and 60 °C. An increase in the conversion was expected as the temperature rises due to the Arrhenius expression (where k = A·exp(−E_a_/RT)). In contrast, the obtained results showed that the increase in the reaction temperature leads to lower FA conversion values. It is assumed that at higher temperatures the favorable reaction is the one producing CO, which may poison the catalyst, and according to the literature, Pd-based catalysts are deactivated by the production of CO [35]. As a result, catalyst’s activity will decrease, as well as the conversion of the reaction. Moreover, Sanchez et al. [34] showed in the past that the possible cause of the deactivation of this catalyst might be the production of CO, and with this theoretical study, we aimed to improve the deactivation. Kosider et al. [36] investigated the FA decomposition in a batch reaction using a Pd/C catalyst showing similar trend in the results as our findings. Their results showed a decrease in FA conversion with an increase in temperature. The activity of the catalyst was found to be lower at higher temperatures as well, revealing the poisoning and deactivation of the catalyst from the production of CO.

**Figure 2:**
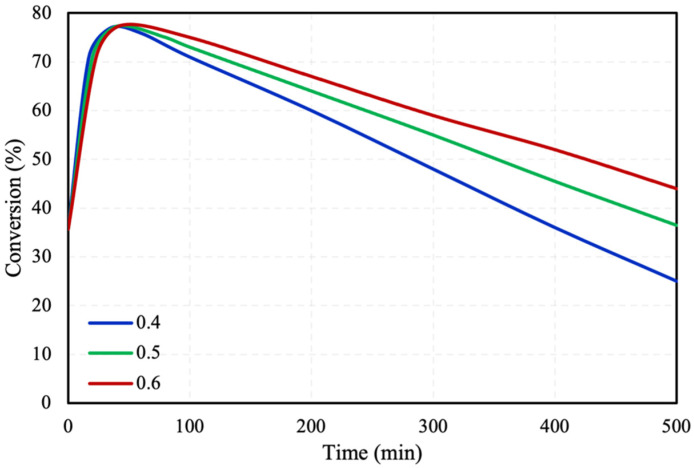
Catalyst porosity effect on FA conversion in packed bed microreactor.

Figure 2 shows the catalyst porosity effect on the reaction’s conversion. Three different porosities of the catalyst were studied, 0.4, 0.5, and 0.6, at 30 °C, achieving the same conversion at the first 50 min approximately of the reaction. After the first 50 min of the reaction, it can be observed from the figure that there is a decrease in the conversion due to the poisoning of the catalyst since the activity of the catalyst is decreased because of the generation of CO. Moreover, higher the porosity of the catalyst, better the performance and the conversion of FA within the microreactor. This can be attributed to the fact that at higher catalyst porosities, the distribution of active sites on the surface of the catalyst could be higher, therefore leading to the increase in the adsorption of FA.

**Figure 3:**
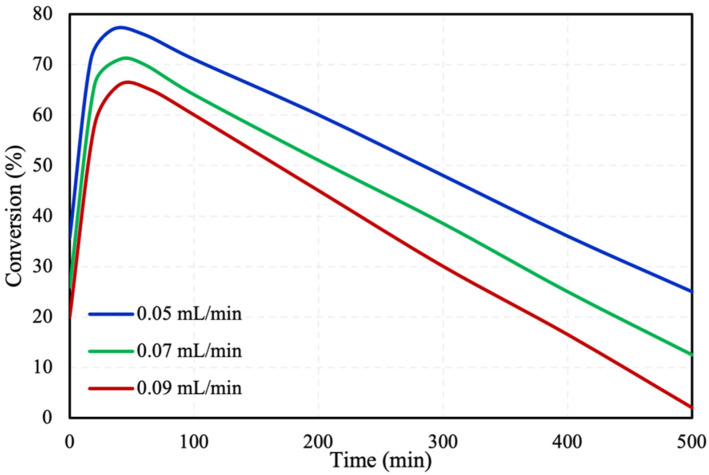
FA initial flow rate effect on FA conversion in packed bed microreactor.

**Figure 4:**
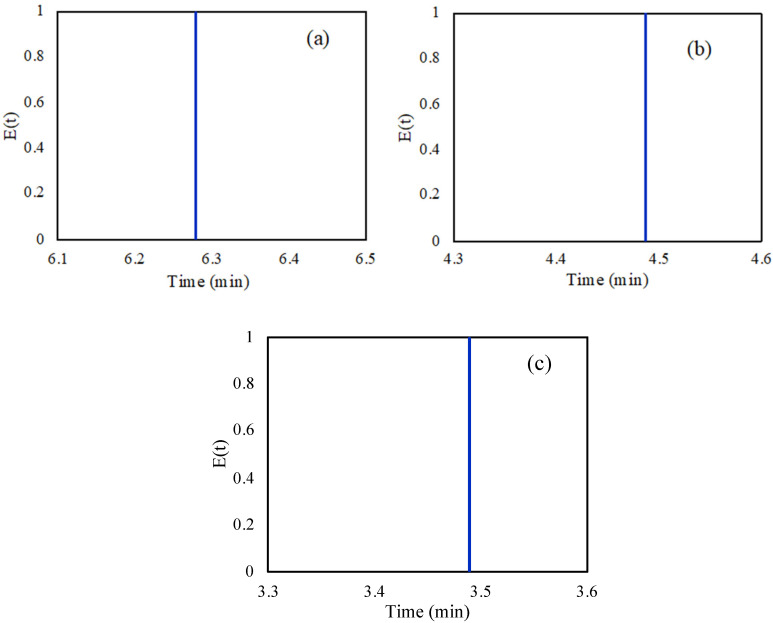
Residence time distribution at (**a**) flow rate of 0.05 mL/min, (**b**) 0.07 mL/min, and (**c**) 0.09 mL/min in the packed bed microreactor.

The FA inlet flow rate was investigated for its effect on the conversion. Three different inlet flow rates were studied (i.e., 0.05, 0.07, and 0.09 mL/min) at 30 °C, with the results presented in Figure 3. It can be observed from the figure that with lower inlet flow rates, higher FA conversions are achieved. By increasing the inlet flow rate, the velocity of the fluid also increased leading to lower fluid residence time inside the microreactor and herein lower conversion. The Particle Tracing Module offered by COMSOL was used in order to calculate the fluid residence time inside the microreactor. Figure 4 presents the distribution of residence time of the fluids inside the microreactor at the three investigated flow rates. It is observed that the residence time of the flow rates of 0.05, 0.07, and 0.09 mL/min are 6.28, 4.49, and 3.49 min, respectively. The figures reveal that at higher flow rates the fluid residence time inside the microreactor is lower, leading to lower FA conversions.

### 2.2. Single and Double Membrane Packed Bed Microreactor

**Figure 5:**
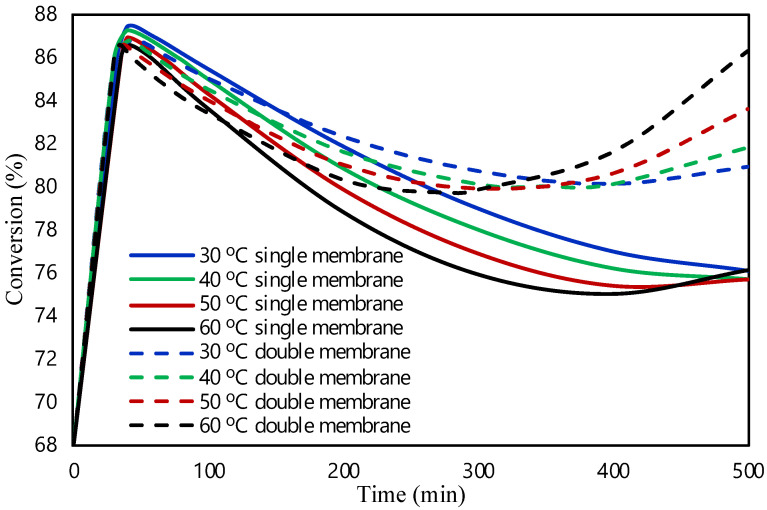
Comparison between the single and double membrane configurations on the formic acid conversion at different temperatures.

In this section, the single and double membrane packed bed microreactor were investigated to improve the performance of the microreactor and hence the conversion of FA using the commercial Pd/C catalyst. The membrane was introduced in the model to selectively remove and separate the CO from the other gas fluids and to avoid the catalyst poisoning. Figure 5 presents the comparison of the conversion of FA within the single and double membrane configurations at different reaction temperatures. The single membrane is selectively removing the CO from the one side of the microreactor (see reactor configurations in Materials and Methods Section 3) while the removal of CO is enhanced at the double membrane microreactor where the CO is removed from both sides. It was also observed that the double membrane microreactor showed better performance than the single due to the more efficient removal of CO from the microreactor. The selective removal of CO prevented and minimized the deactivation of the catalyst with respect to time, and herein, the performance and conversion of the microreactor was improved. Lastly, it was revealed in the double membrane configuration that by removing CO from the system, the conversion tends to increase, while increasing the temperature, the deactivation of the catalyst is minimized. Hafeez et al. [27] showed that the removal of CO using a single membrane microreactor inhibits and minimizes the deactivation of the catalyst. Sandoval and Gigola [37] revealed through the TPD profile that the adsorption of CO from the surface of the active sites changes from the favored multiple coordinated strong form to the weak, indicating that species such as the CO tend to desorb at higher temperatures (>60 °C). Therefore, the simultaneous combination of the efficient removal of CO using membranes and the removal of the adsorbed CO from the active sites as the temperature of the reactor increases is responsible for the enhanced stability of the catalyst.

**Figure 6:**
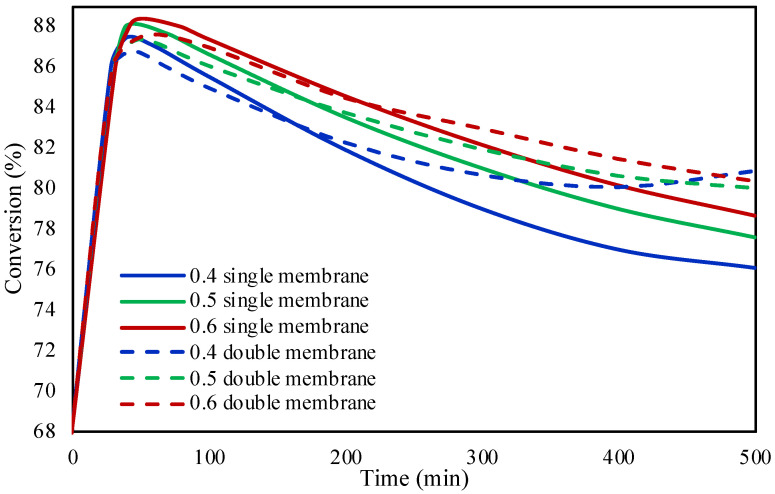
Comparison between the single and double membrane configurations on the formic acid conversion at different catalyst porosities.

Figure 6 shows the effect of catalyst’s porosity on FA conversion in both membrane configurations at 30 °C. The three different investigated catalyst porosities of 0.4, 0.5, and 0.6 showed similar trend as the investigation in the packed bed microreactor. The conversion during the first 40 min is similar, while due to the poisoning of the catalyst the conversion decreases thereafter. The membrane configurations showed an increase in the conversion while increasing the catalyst porosity, likewise in the case of packed bed microreactor. The double membrane microreactor due to better removal efficiency of CO achieved slightly higher conversions of FA in comparison to the single membrane microreactor.

**Figure 7:**
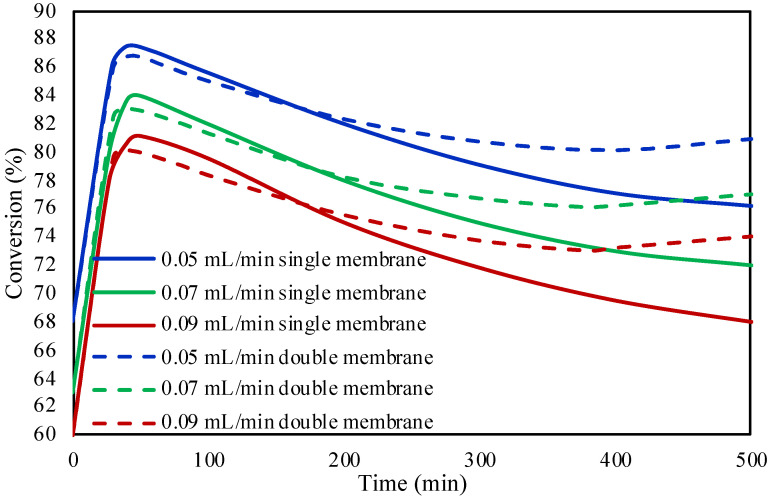
Comparison between the single and double membrane configurations on the formic acid conversion at different inlet flow rates.

The investigation of the flow rate’s effect on the FA conversion, at 30 °C, in both single and double membrane microreactors (Figure 7) showed similar results as the packed bed microreactor. The single membrane microreactor is selectively removing CO from the one side of the microreactor (see reactor configurations in Materials and Methods Section 3) while the double membrane microreactor is removing the CO from both sides of the reactor. The three different flow rates of 0.05, 0.07, and 0.09 mL/min revealed that the increase in the inlet flow rate is leading to lower FA conversions. Similar to the packed bed microreactor investigation, the FA conversion according to the flow rate effect is attributed to the residence time of the fluid inside the microreactor. It was also observed that the efficient removal of CO in the double membrane configuration increased the conversion of FA with respect to time.

**Figure 8:**
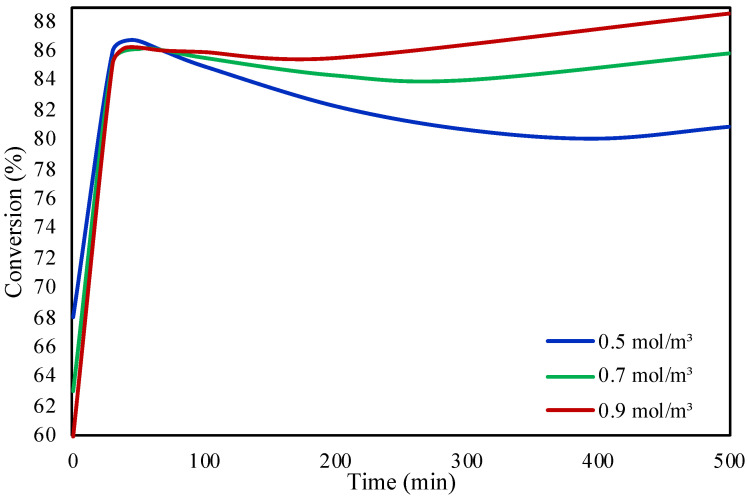
FA concentration effect on the conversion of FA in the double membrane microreactor.

In this work, we also aimed to investigate the influence of FA concentration on the conversion in the double membrane microreactor. Three different FA concentrations were studied, 0.5, 0.7, and 0.9 mol/m^3^ at 30 °C. Figure 8 presents the concentration effect of FA on the reaction conversion. It was observed that by increasing the FA concentration higher conversions are achieved with respect to time. The increase in the concentration leads to higher reaction rates as there is a correlation between the reaction rate and the FA concentration according to the reaction rate.

## 3. Materials and Methods

### 3.1. Modelling Methodology

In this study, 5 wt.% Pd/C catalyst was used under mild conditions in a packed bed, single membrane, and double membrane microreactors in 2D models for the FA decomposition. A 0.05 mL/min flow rate was used for this study, and the reaction was studied from 30 to 60 °C. CFD modelling studies were implemented to show the behavior between the heterogeneous reaction of particles and fluid within these microreactors. The validity of the model was investigated in a previous work revealing the validity of the designed model [20].

**Figure 9:**
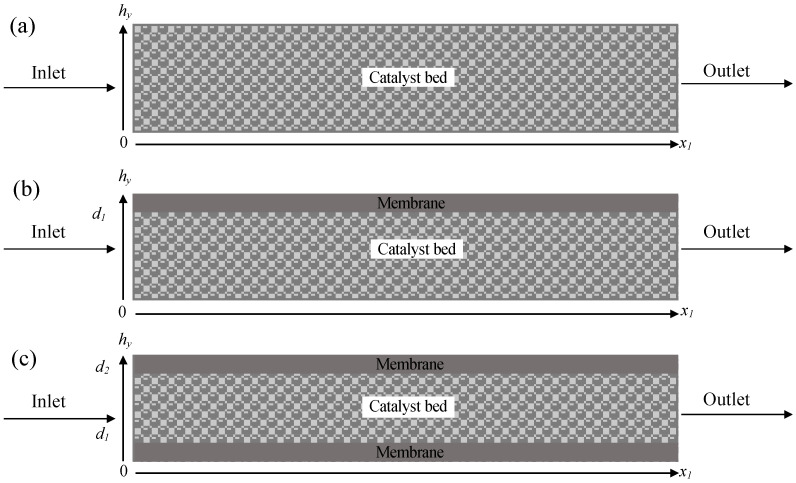
Reactor configurations of (**a**) packed bed, (**b**) single membrane, and (**c**) double membrane microreactors used for this study.

The microreactor configurations were designed as 2D models (Figure 9) hypothesizing that temperature, flow, and mass profiles take place only in the axial and radial directions. The assumptions with which the model was designed include: (i) laminal flow and unsteady state conditions were applied, (ii) only CO can pass through the membrane or membranes, (iii) there are no heat transfer phenomena since isothermal conditions were applied, (iv) the liquid dissolution in the gas phase and in the membrane is considered negligible, and (v) the transport coefficient values and physical properties of the constant axial fluid velocity were uniform. Finally, the membrane material for the selective removal of CO is polytetrafluoroethylene (PTFE).

### 3.2. Reaction Kinetics

The commercial Pd/C catalyst used for the computational investigation was previously examined exhibiting selectivity for H_2_ of 99.9% and a TOF value of 1136 h^−1^ at 30 °C for FA decomposition to H_2_ and CO_2_ [34]. The negligible amount of CO that is produced is significant in order to poison and deactivate the catalyst. The rate of reaction can be expressed as:

(3) $$r=k\times C^{n}$$

where k and r are the reaction constant and reaction rate, respectively. C is the FA concentration, and *n* is the reaction order [34].

The activity parameter (*a*(*t*)) is introduced in the model in order to predict the catalyst activity. This parameter is correlated to the concentration of CO, which is accumulated in the microreactor and poisons the catalyst. The catalyst deactivation and herein the decrease in the conversion is modelled based on concentration of CO with the activity parameter given as:

(4) $$a\left(t\right)=1-\lambda \times C_{CO}$$

### 3.3. Conservation Equations

The equation of the transportation of the species in the catalyst bed is given as:

(5) $$u_{x}\frac{\delta c_{i}}{\delta x}{=D}_{i,A}\frac{\delta^{2}c_{i}}{\delta x^{2}}+D_{i,T}\frac{\delta^{2}c_{i}}{\delta y^{2}}-J_{i}S_{b}$$

where $u_{x}$ (m/s) is fluid’s velocity in x direction, $D_{i}$ and $J_{i}$ are the coefficient of axial dispersion in the axial and transverse directions and the fluid’s molar flux in the catalyst, respectively, and $S_{b}$ is the active specific surface area of particles that is in contact with the reactant components in the catalyst bed and is expressed as:

(6) $$S_{b}=S_{a}\left(1-\varepsilon \right)$$

where ε and *S_a_* are the catalyst bed voidage and the catalyst specific surface area (m), respectively [38]. The specific surface area of spherical particles is:

(7) $$S_{a}=\frac{3}{r_{pe}}$$

where *r_pe_* (m) is the catalyst’s particle radius.

At the interface of pellet–fluid, the film condition’s assumption is made, considering the external mass transfer as a resistance described as:

(8) $$J_{i}=h_{i}\left(c_{i}-c_{i,ps}\right)$$

(9) $$h_{i}=\frac{Sh\cdot D_{i}}{{2r}_{pe}}$$

(10) $$Sc=\frac{\mu }{\rho \cdot D_{i}}$$

(11) $$Re=\frac{{2r}_{p}\cdot \rho \cdot u_{x}}{\mu }$$

(12) $$Sh=2+0.552{Re}^{1/2}{Sc}^{1/3}$$

where $h_{i}$ and $c_{i,ps}$ are the coefficient of external mass transfer and species concentration at catalyst’s surface. Sc, ρ, and μ are the Schmidt number, the fluid’s density, and viscosity, respectively. Re and Sh are the Reynolds number and the Sherwood number, respectively [39,40].

The reaction is occurring within the catalyst particle combining the feature of Reactive Pellet Bed on COMSOL. Across the spherical shell mass balance of the powdered particle, an extra 1D predefined dimension on the normalized radius $\left(r=r_{dim}/r_{pe}\right)$ is expressed as:

(13) $$4\pi N\left\{r^{2}r_{pe}^{2}\varepsilon_{pe}\frac{{\partial c}_{pe,i}}{\partial t}+\nabla \cdot \left(-r^{2}D_{i,eff}\nabla c_{pe,i}\right)=r^{2}r_{pe}^{2}R_{pe}\right\}$$

where N is particles number, $D_{i,eff}$ is the coefficient of effective diffusion of fluids in the pores of powdered particle, $c_{pe,i}$ and $R_{pe}$ are the concentration of components in the powdered catalyst and the reaction rate term, respectively.

The diffusion coefficients (effective) of species in the particle material are described by the Knudsen or bulk diffusivity expressed as [39]:

(14) $$D_{i,eff}=\frac{D_{i,AB}\Phi_{p}\sigma_{c}}{\tau }$$

where $D_{i,AB}$ is the diffusivity of fluid components in bulk, $\Phi_{p}$ is the porosity of the powdered catalyst, and $\sigma_{c}$ and τ are the constriction factor and tortuosity, respectively.

The transportation of components through the membrane is described as:

(15) $$D_{i,m}\left(\frac{{\delta^{2}c}_{i,m}}{{\delta x}^{2}}+\frac{\delta^{2}c_{i,m}}{{\delta y}^{2}}\right)=0$$

where $c_{i,m}$ and $D_{i,m}$ are the concentration and the species coefficient of diffusion in the membrane.

The Particle Tracing Module is a tool offered by COMSOL to decipher the distribution of residence time by computing the direction of particles. The residence time distribution is an alternative to the first-order Newtonian formulation given as:

(16) $$\frac{dq}{dt}=v$$

where q is the pellet position (m), and v the velocity of the particle (m/s).

The boundary conditions used for investigated microreactor configurations are given as:

for packed bed microreactor,

(17) $$\mathrm{at}\;x=0;\;c_{i}=c_{i,in}$$

(18) $$\mathrm{at}\;x=x_{i};\;\frac{\delta c_{i}}{\delta x}=0$$

(19) $$\mathrm{at}\;y=0;\;c_{i}=0\;\;\;$$

(20) $$\mathrm{at}\;r=1;\;c_{i,p}=c_{i,ps}$$

(21) $$\mathrm{at}\;r=0;\;\frac{\delta c_{i,p}}{\delta r}=0$$

for single membrane microreactor,

(22) $$\mathrm{at}\;x=0;\;c_{i}=c_{i,in}$$

(23) $$\mathrm{at}\;x=x_{i};\;\frac{\delta c_{i}}{\delta x}=0$$

(24) $$\mathrm{at}\;y=0;\;c_{i}=0\;\;\;$$

(25) $$\mathrm{at}\;r=1;\;c_{i,p}=c_{i,ps}$$

(26) $$\mathrm{at}\;r=0;\;\frac{\delta c_{i,p}}{\delta r}=0$$

(27) $$\mathrm{at}\;y=d_{1};\;c_{i,m}=Hc_{i}$$

(28) $$\mathrm{at}\;y=h_{y};\;c_{i,m}=c_{i,g}\;\;\;\;$$

and for double membrane microreactor,

(29) $$\mathrm{at}\;x=0;\;c_{i}=c_{i,in}$$

(30) $$\mathrm{at}\;x=x_{i};\;\frac{\delta c_{i}}{\delta x}=0$$

(31) $$\mathrm{at}\;y=0;\;c_{i}=0\;\;\;$$

(32) $$\mathrm{at}\;r=1;\;c_{i,p}=c_{i,ps}$$

(33) $$\mathrm{at}\;r=0;\;\frac{\delta c_{i,p}}{\delta r}=0$$

(34) $$\mathrm{at}\;y=d_{1},\;\;y=d_{2};\;c_{i,m}=Hc_{i}$$

(35) $$\mathrm{at}\;y=0,\;\;y=h_{y};\;c_{i,m}=c_{i,g}$$

COMSOL Multiphysics 6.0 was used for this investigation coupling the conservation, mass balance equations, and boundary conditions on a laptop with 32 GB of RAM. A sensitivity analysis of the mesh was performed to study the validity of the CFD study by varying the mesh size. Table 1 shows the mesh sensitivity analysis results varying the mesh quality. It was obtained that the results were mesh independent at all three microreactor configurations as the solution was checked for higher degrees of freedom and the difference between the results was found to be less than 1%. Table 2 consists all the modelling parameters that were used for this study.

## 4. Conclusions

In this work, three different microreactor configurations were studied for their performance on FA dehydrogenation using 5 wt.% Pd/C catalyst. This study is offering for the first time a substantial solution for minimizing the poisoning of the catalyst by introducing membranes into the reactor system. The innovative implementation of CO selective membranes allows the removal of CO inhibiting the deactivation of the catalyst. The packed bed microreactor showed good performance only in the first 50 min, at all the investigated temperatures, as the generation of CO poisoned the catalyst and reduced its activity. Also, it was observed that the production of CO is more favorable at higher temperatures as the catalyst deactivation is increased. Main aim of the work was to incorporate a membrane in the microreactor system in order to remove the CO and avoid the catalyst deactivation. The single and double membrane configurations showed better performance in terms of lowering the deactivation of the catalyst with the last one having the best performance. Parametric studies investigating the effect of catalyst porosity on the conversion of FA was performed for all three microreactor configurations, revealing that higher the catalyst porosity is, higher is the conversion, due to higher number of active sites and more enhanced adsorption of FA on the catalyst. The effect of the inlet flow rate of FA showed that lower flow rates tend to have higher conversion rates due to the higher residence time of the fluids inside the microreactor. It also investigated the concentration of FA effect on the conversion in the double membrane microreactor showing that the increase in conversion leads to higher reaction rates according to the rate of the reaction. The efficient removal of CO in the double membrane microreactor had a FA conversion of over 80% in all case studies. Additional 3D studies considering the veritableness of the performance of the microreactors will be performed as future work in order to obtain substantial results. Moreover, the efficient removal of CO by using the specific reactor configurations can be useful as possible solutions for other chemical processes.

## Figures and Tables

**Table 1:** Mesh sensitivity analysis of the microreactor configurations used in this study.

Mesh Quality	Time to Compute	Elements	Vertices	Boundary Elements	Degrees of Freedom	Max. Conv. Rate (30 min)	Difference
Normal	5 s	98	68	36	996	77.766%	Reference
Fine	5 s	154	100	44	1524	77.752%	−0.0018%
Extra Fine	10 s	1010	564	116	9444	77.720%	−0.041%
Extremely Fine	23 s	4030	2132	232	36,972	77.703%	−0.022%

**Table 2:** Parameters used for the CFD modelling study.

Symbol	Value	Units	Description
$c_{FA}$	0.5	mol m^−3^	FA inlet concentration
u	0.05	mL min^−1^	Volumetric inlet flow rate
$x_{i}$	25	mm	Microreactor length
$y_{i}$	4	mm	Microreactor height
$d_{m}$	1	mm	Membrane thickness
Τ	303–333	K	Reaction temperature
$d_{pe}$	4	nm	Catalyst pellet size
ρ	1	kg m^−3^	Fluid density
$\rho_{b}$	1300	kg m^−3^	Catalyst density
$\varepsilon_{0}$	0.4	-	Clean catalyst porosity
R	8.314	J mol^−1^ K^−1^	Ideal gas constant

## Data Availability

Data available on request.
